# Mechanical Dentistry—Its Present Condition. &c.

**Published:** 1876-07

**Authors:** L. P. Haskell


AMERICAN JOURNAL

OF

### DENTAL SCIENCE.

Vol. X. THIRD SERIES—JULY, 1876. No. 3.

### ARTICLE I.

### *Mechanical Dentistry: Its Present Condition, and the Advantages of Celluloid in Placing it upon a True Artistic Basis.*

BY DR. L. P. HASKELL.

Read before the Wisconsin State Dental Society.

Having been invited by your Committee to prepare a paper for this meeting of your society, upon the subject of Celluloid, I have ventured to change the subject somewhat and have selected for my theme a few practical remarks on "Mechanical Dentistry: its present condition, and the advantages of Celluloid in placing it upon a true artistic basis."

Notwithstanding all the improvements that have been made in materials for artificial dentures within the past twenty five years, but little advance has been made among the profession generally in appropriating these materials so as to secure the best results in an artistic manner. And we still witness the same stilted uniformity in arrangement of teeth, reminding one of strings of white beans; the same puffed out lips, looking as though a wad of cotton were placed under the lip; the same depression that inevitably follows the extraction of the eye teeth, with no effort to restore this important facial expression; the same "horse-shoe" contour of the arrangement of the crowns of the teeth, and so unlike nature.

Now, gentlemen, with all that is being done to improve the operative branch of dental science, would it not be well to devote a little more time to the improvement of this long neglected branch of the science, and arouse the members of the profession to the necessity of redeeming it from the disrepute into which it has been brought by the use of vulcanized rubber in the hands of the abominable quacks who disgrace the name of "dentist," to say nothing of the term "doctor," they so delight to affix to their names, it being in fact their only stock in trade.

A better illustration of this class of men, these "hangers-on" to the profession, could not be found than in the remark made by one of them, a resident of your State, when he said, "any man who could make a hoe-handle, could make a set of teeth;" and surely those he made were of the hoe-handle order.

I am well aware there is but little satisfaction in devoting the time necessary to the perfecting of an artificial denture so as to realize all that it is possible to realize in the restoration of the features, and the adaptation of the teeth to the individual in appearance and usefulness, on account of the necessity of competing with the aforesaid quacks, who so infest the community. For by the introduction of rubber and "gum sections," a man with but little knowledge of dentistry, can make a so-called set of teeth. Consequently as operative dentistry is so much more remunerative, the temptation is great to advise the patient to have the rubber set, and then submit the construction of it to the student, or employe, who often knows but little more than the student, and never would be able to exercise taste or skill should he live a thousand years.

But the most singular feature of this whole business is that so many people are willing to wear such abortions, and people too, who in other matters exercise taste and judgment. And the first thing to be done is to educate the community to an appreciation of something better than is produced by these "hoe-handle tooth carpenters." Show them that there is nothing worn or used about the person of more importance than the set of artificial teeth. Remind them that in seclecting a dress, a hat, or a pair of boots, an article of jewelry, they do not select it because it is *cheap*, but because it is adapted to their individuality, and if it is true of these things, how much more is it true of that which applies to the features. The practiced eye can discern very often at a glance the victim of false teeth, without the patient opening the mouth, there is such a peculiar expression produced. In truth so common are these artificial substitutes that this expression is becoming almost a *national* one, and must sooner or later attract the attention of foreigners as a marked peculiarity of our American race.

The patient should be made to realize how utterly impossible it is for the dentist unless suitably remunerated, to devote the time necessary to a suitable selection of teeth for each case; the proper arrangement of the same by the mouth, which is indispensable, and *never* to rely upon an articulator, which is merely a preliminary guide, and then to the "filling out" so as to restore the lost expression, securing as near a perfect result as possible, and so near to nature as to deceive even the practiced eye.

If people would exercise a little sense in these matters they would cease to patronize "hoe-handle" tooth carpenters and $8 quacks, and before employing a dentist ascertain if possible if he be thoroughly competent to undertake what he professes.

The proper and thorough education of the dental student in mechanical dentistry is of the first importance; in this respect our dental colleges are greatly deficient, a large proportion of the younger members of the profession are unable to make a respectable gold denture, and as to " continuous gums " that is utterly out of the question, and so are obliged to confine their efforts to vulcanized rubber thrown together after the usual routine method, and of course are obliged to tell their patients that this material is the best and suitable for all cases.

It is all right that the student should be educated to the very highest degree to the importance of saving the natural organs, this is the first and most important duty in his profession; but your duty is only partly done if you do not fully instruct him how to restore by art the lost contour of the face, and secure to the unfortunate patient all that is possible in the insertion of the set of artificial teeth.

Better results would be accomplished if a greater number devoted themselves to this branch of the profession, for it is equally true in dentistry as in the practice of medicine, that greater perfection can be reached through specialties than by endeavoring to compass the whole. This would be practicable however only in the larger towns.

Let us go back a few years to the days of metal plates and " single " teeth, gum or plain. It requires at least mechanical skill to construct a set of teeth, although little could be done in restoring the features, or securing a natural arrangement of the teeth in all respects. Still there was room for a display of skill and taste, even with these materials.

The introduction of carved blocks was a great advance in the right direction, yet in the use of these it was often necessary, in order to secure the best results that the carver should see the patient and arrange his own waxes.

Next in order came the greatest boon ever conferred upon the victim to false teeth (and would that more appreciated it) the introduction of Allen's Continuous Gum. This, in the hands of the expert, is perfection itself. And I can safely say of it, after twenty-three years use, that for strength, durability and perfect imitation of nature, there is nothing that approaches it, and in truth nothing can be said against it. But this again requires skill, careful manipulation and experience in order to accomplish the best results. This work has also often fallen into the hands of slip-shod, incapable men, who have brought it into disrepute. And the dental colleges fail to instruct fully their students in its manipulation.

Then came vulcanite, which has proved both a blessing and a curse—a blessing in that it has enabled many to wear false teeth, who would otherwise been unable to do so—a curse, in the first place, in that it has enable the aforesaid "hoe-handle" fraternity to foist themselves upon the community, and secondly, that it is so often recommended and used, when gold or other metal would be far preferable. This material, however great its advantages in some respects, is very objectionable in others, not the least of which is its color, and then no one who has any experience in its use can have failed to witness its pernicious effects upon the mucous membrane on many mouths. But last, though not the least of its objectionable features, is found in the fact that the case with which it is manipulated is a great inducement for the dentist to make use of it in cases where other material would be preferable; and he is unable to avoid the more laborious and complicated method by resorting to this. But vulcanized rubber has had its day, and the time is not far distant when it will be numbered among the things of the past. Peace to its ashes, and may Josiah retire to private life, never more to vex the soul of the poor dentist with his bogus patents.

And this brings me to the consideration of the greatest improvement in mechanical dentistry, since the introduction of Allen's continuous gum, namely, *celluloid*. We have not a new material, but new in its most recent developments. Its base, *gum cotton*, has been used many years in different forms. "Rose Pearl," in the hands of a few, met with some degree of success, the credit of which, I believe is due to Dr. McClelland, of Louisville. But this had its ob jectionable features. Another development of this base was the so-called "pyroxolene." This bid fair to be quite successful for a time, but alas it too had its fatal errors, and like rose pearl, failed not so much from the nature of the material as from the mode of manipulation. I have a case of this in wear for nearly five years, and it still is in good condition. But after many years of investigation and experiment, the material has been developed into its present condition, and has been used long enough to establish its claim to real genuine advance in material for plates, and a gum, too, for artificial teeth. Doubtless other improvements will be made in the material as well as in its manipulation. One of the most important of these already made, is in the use of flax fibre instead of cotton. Then its manufacture in large quantities and with improved machinery makes it more uniform in its texture. The substitution of steam heat for oil, and now *dry heat*, instead of steam, seems to secure still better results.

We have in this material *all* the good points of rubber, some that *it* has not, and I think *none* of its objectionable features. It is not suited to all cases, and there is no one material that is. It is stronger than rubber, more easily worked, more cleanly to work, packs tightly around the teeth, and with its near resemblance to flesh, enables one with an artistic taste to produce most desirable results, in combination with plain teeth. Of course, the dentist who wishes to avoid *extra labor* will still use the gum-sections in all cases. If I used them, should charge less rather than more than for the plain teeth, for with me "time is money."

This material approaches the nearest to a substitute for continuous gum that has ever been devised, and yet it is very far from being a complete substitute. It is impossible of course to secure the delicate shadings of the natural gums so perfectly obtained in the porcelain, and lacks the strength and durability of the former base.

No one entering the dental profession should consider himself thoroughly qualified for it, until he can construct a set or part of a set of teeth, on any base now in use and best adapted to such case; and he should adopt as a rule of practice the principle that no material is suitable for every case he is called upon to insert. In the application of the various materials my rule is to recommend according to the circumstances of the case. If the patient is able to pay for the best, and will appreciate it nothing has ever so fully met all the requirements of the case as Allen's continuous gum *for full sets.* Next to this, gold plate with either the rubber or celluloid attachments is preferable because the plate is much thinner and stronger, and in close articulations the teeth can be fastened so much stronger. In the partial lower sets I have found for many years the best results secured by the use of rubber, as it is so much more easily adjusted to the peculiar shape of the jaw than a swedged plate, and strengthened by gold across the inside of the front teeth. In many cases where the lower alveolar is very flat, and the muscles and integuments left displace the plate, a cast metal plate is very useful, and next in order to the continuous gum, for in such cases additional weight is very necessary. However, in a large proportion of cases where expense is the principal consideration, the celluloid can be used to great advantage, and will undoubtedly prove the greatest improvement in mechanical dentistry since Allen introduced his continuous gum.

And then in the use of any of these materials the dentist must be governed by the length of the patient's purse, and never undertake any job at a price that will not admit of spending all the time that is necessary to its successful execution, and also to a possible failure and necessity of remaking the case.

Perhaps a few suggestions in regard to the use of gold plate may be of interest to some of the younger members of of the profession, as many find it difficult to secure good fits with this material. I can say that I find it just as easy to secure a good fit and suction with a swedged plate as with rubber or celluloid. And the secret lies in the use of Babbit metal castings, and I seldom use but one cast in any case. As a general thing the Babbit metal of commerce is too soft, as in making it the correct formula is not used. Any one can make it for themselves, as follows: One part copper, two parts antimony, eight parts tin; melt in a black lead crucible in the order named. The result is a very hard, smooth, unshrinking metal, that melts at a low temperature. For the counter die use lead with the addition of tin, about one-sixth turned upon the die as cold as it can be turned. Then by using oiled sand the whole process of dental-die making is greatly simplified, and reduced to a perfect certainty each time, and no difficulty will be found in fitting gold or other metal plates. In making full sets, I use the rubber or the celluloid attachments, but in partial sets generally solder the teeth. It would be far better for the patients in multitudes of cases were gold oftener used, and more would be used were it oftener recommended by the dentist.

Gentlemen, I have endeavored in as few words as possible to call your attention to the condition into which mechanical dentistry has been brought by a class of incompetent men, who are neither artists nor mechanics, and to suggest some means whereby this condition of things can be remedied, and now invite your further consideration of the subject, and trust your society will take some steps to bring about a reform both through the education of the student, and also of the public, to an appreciation of something better than they are so much accustomed to see in their daily walks and *conversations*.

